# Combined Features in Region of Interest for Brain Tumor Segmentation

**DOI:** 10.1007/s10278-022-00602-1

**Published:** 2022-03-15

**Authors:** Salma Alqazzaz, Xianfang Sun, Len DM Nokes, Hong Yang, Yingxia Yang, Ronghua Xu, Yanqiang Zhang, Xin Yang

**Affiliations:** 1 School of Engineering, Cardiff University, Cardiff, CF24 3AA UK; 2 Department of Physics College of Science for Women, Baghdad University, Baghdad, Iraq; 3 School of Computer Science and Informatics, Cardiff University, CF24 3AA Cardiff, UK; 4 Department of Radiology, The Second People’s Hospital of Guangxi Zhuang Autonomous Region, Guilin, 541002 PR China; 5 Department of Radiology, The People’s Hospital of Guangxi Zhuang Autonomous Region, Nanning, 530021 PR China; 6 Centre of Information and Network Management, The People’s Hospital of Guangxi Zhuang Autonomous Region, Nanning, 530021 PR China; 7 State Information Center of China, Beijing, 100045 PR China

**Keywords:** Brain tumor segmentation, Multi-modal MRI, Convolutional neural networks, Gray-level co-occurrence matrix, Decision tree

## Abstract

Diagnosis of brain tumor gliomas is a challenging task in medical image analysis due to its complexity, the less regularity of tumor structures, and the diversity of tissue textures and shapes. Semantic segmentation approaches using deep learning have consistently outperformed the previous methods in this challenging task. However, deep learning is insufficient to provide the required local features related to tissue texture changes due to tumor growth. This paper designs a hybrid method arising from this need, which incorporates machine-learned and hand-crafted features. A semantic segmentation network (SegNet) is used to generate the machine-learned features, while the grey-level co-occurrence matrix (GLCM)-based texture features construct the hand-crafted features. In addition, the proposed approach only takes the region of interest (ROI), which represents the extension of the complete tumor structure, as input, and suppresses the intensity of other irrelevant area. A decision tree (DT) is used to classify the pixels of ROI MRI images into different parts of tumors, i.e. edema, necrosis and enhanced tumor. The method was evaluated on BRATS 2017 dataset. The results demonstrate that the proposed model provides promising segmentation in brain tumor structure. The F-measures for automatic brain tumor segmentation against ground truth are 0.98, 0.75 and 0.69 for whole tumor, core and enhanced tumor, respectively.

## Introduction

The growth of abnormal and uncontrolled cells inside the brain or spinal canal is defined as brain tumor. There are four main types of the primary brain tumors: gliomas, meningiomas, pituitary adenomas and nerve sheath tumors. In biomedical analysis, segmentation of brain tumors from multi-modal magnetic resonance imaging (MRI) plays a vital role. Glioma can appear anywhere in the brain with different shapes, and there is a large variety and high complexity within one type of tumors in terms of intensities and textures [2]. Therefore, the challenge is to develop a method which creates a precise segmentation and works for multiple tumor classes and different imaging equipment [1].

Early detection and localization of the tumors can lead to changes in patient treatment plan that will impact on his/her health outcomes. Several approaches have been suggested in the literature for detection and segmentation of tumors in MRI modalities [3]. Some are based on the extracted features from the MR images, and they design models [4]. Hand crafted features were used in different brain tumor segmentation techniques which are fed into a classifier such as a decision tree (DT) [5]. The DT classifier demonstrated the best results among different conventional classifiers [4]. The limitation of the approaches based on hand designed features is that these methods require a large number of features for best representation of the brain tumor tissues. As a result, they need a high dimensional size of data which require more computational time for processing and a large number of experiments to optimize the parameters of the classifier. To address these problems, many deep learning-based methods were developed recently, which provide better accuracy for brain tumor segmentation [6–8]. Ghaffari et al. [24] proposed a 3D CNN model based on a variant of the U-Net architecture with some modifications to obtain the local features. They utilised connected component analysis as post-processing step to enhance the performance. In the paper [25], an efficient cascade CNN model was implemented for extracting both local and global features in two different ways with different sizes of extraction patches. Daimary et al. [26] demonstrated an algorithm that contains three hybrid CNN models, U-SegNet, Res-SegNet, and Seg-UNet, which are designed for high-accuracy automatic segmentation of brain tumor from MRI images. The suggested models inherit attributes from the most common CNN models for semantic segmentation, SegNet, U-Net, and ResNet. However, using only a method that is based on deep learning is insufficient for performing an accurate brain tumor region segmentation. The limitation is that the local features related to the changes of the texture tissue due to tumor growth are not sufficiently considered in the SegNet-based approach [22]. At the same time, some hand-crafted feature extraction methods take into account the local dependencies of the pixel classes, such as grey-level co-occurrence matrix (GLCM)-based texture features [9]. The GLCM was claimed to be the most popular texture-based method for MR images [10].

The motivation of this paper is to develop a hybrid method arising from the need of high accuracy segmentation. We proposed a new learning-based method, which combines the machine learned features and the hand crafted features for automated segmentation of the brain tumor structures from the generated ROI images from MRI dataset. The machine-learned features are the score maps extracted from the de-convolution layer in the trained SegNet network, and the hand-crafted features are the GLCM-based texture features. The proposed method was applied and evaluated on the publicly available BRATS 2017 dataset [4, 11].

The main contributions of this paper are as follows:

- An automatic method is proposed to generate region of interest (ROI) segment which is in agreement with experts’ delineation across all grades of gliomas through using a single commonly used MRI protocol, i.e. FLAIR as input data.
- A DT classifier is applied only to the pixels that are considered as the tissues of ROI, which helps to largely reduce the computational cost through reducing the data size for classification.
- A novel method is developed to overcome the limitation of SegNet network and increase the performance of detecting necrosis and enhanced brain tumor regions by combining hand-crafted features with machine-learned features.

## Proposed Approach

**Fig. 1:**
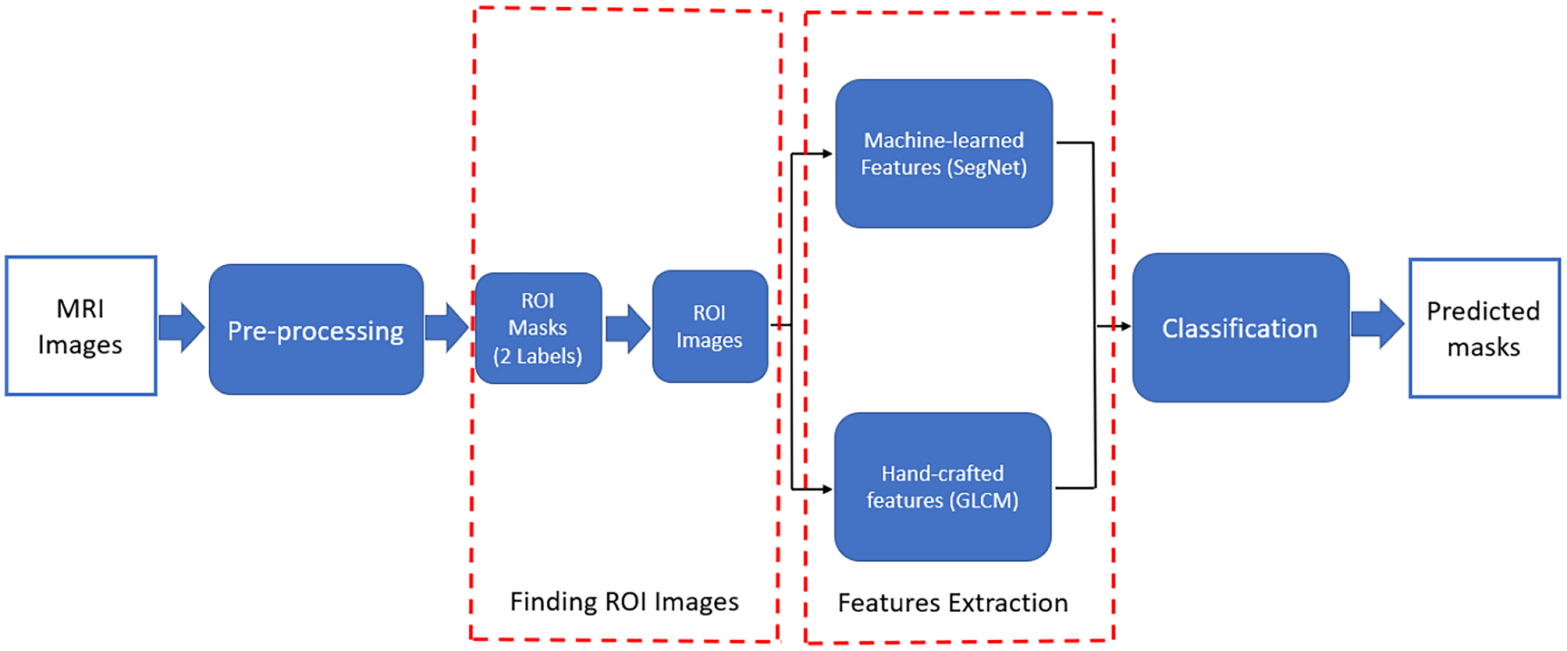
Pipeline of the brain tumor segmentation

The proposed segmentation method includes four main steps: pre-processing, ROI image generation, feature extraction, and pixel classification. The pre-processing is first performed through removing the artefacts and normalizing the intensity ranges of the MR images. Then, a binary mask as the ROI containing only tumor tissues is identified with a SegNet model trained on a single MRI modality, and the mask is applied on all the MRI modalities to produce ROI images. The machine-learned features are extracted for each pixel using another SegNet model trained on the ROI images, and the hand-crafted texture features are calculated based on GLCM. Finally, the combined features are fed into a DT classifier for labelling the pixels to corresponding tissues. The whole pipeline of the proposed method is shown in Figure 1. In the rest of the paper, we call our method as SegNet_GLCM_DT for short.

### Data Pre-processing

Artefacts often exist in MRI data due to the inhomogeneity in the magnetic field or the patient’s small movements during the scan period. As a result, a bias is produced across the results of the scans which affects the accuracy performance of the segmentation results especially when the segmentation is made by a computer-based algorithm. To correct that, we applied N4ITK bias field correction to all MRI modalities to remove unwanted artefacts [20].

Since the intensity values across MRI slices vary greatly, additional normalization step is also applied by subtracting the mean and dividing by the standard deviation of the brain region. Additionally, removing the top and bottom 1% intensity values in the normalization process brings them within a coherent range across all images in the training phase. To remove a significant portion of unnecessary zeros in the MRI dataset and to save training time with huge reduction of memory requirements for 3D data sets, we trimmed some black parts of the image background from the data of all modalities to get input images of size ${192\times 192}$.

### Region of Interest Image Generation

The management of radiation dose planning and treatment response monitoring comes from the accurate detection of the tumor extent structure. Additionally, delineation of the tumor region, which is considered as ROI, is important for assessing the growth of glioma grades as well as extracting image features from abnormal regions for further tumor classification [12].

**Fig. 2:**
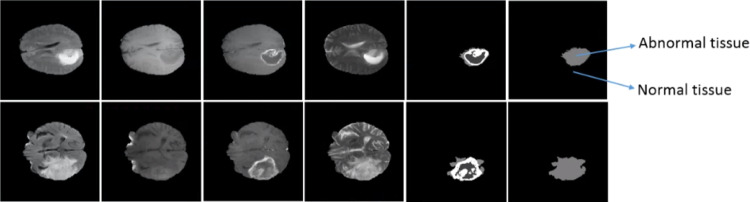
(left to right), FLAIR, T1, T1ce and T2 MRI modalities, ground truth with four classes and ground truth with two classes

In this paper, an initial ROI was firstly identified using a semantic segmentation network, the SegNet [21]. A pre-trained SegNet model was modified and trained with each MRI modality as input separately for binary segmentation (normal and abnormal tissues). This process involves two main steps: ROI mask detection and ROI MRI image generation. An important scenario for this binary segmentation is to prepare ground truth masks by converting the original ground truth with four labels into that with two labels. See Fig. 2.

**Fig. 3:**
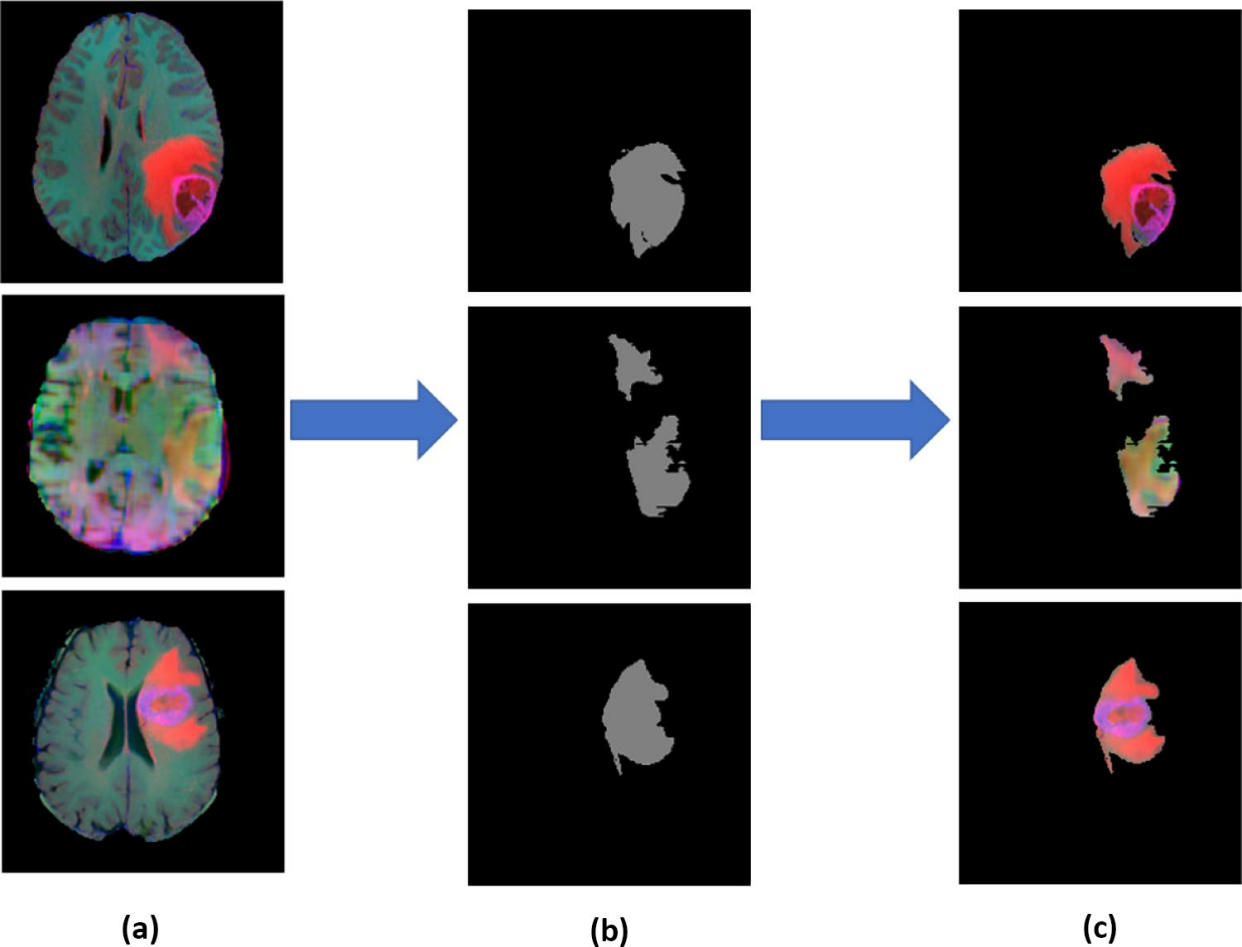
(**a**) Combined MRI modalities, (**b**) ROI masks Images and (**c**) MRI ROI images

To obtain optimal ROI mask, the pre-trained SegNet network was fine-tuned separately for each MRI modality. The four trained SegNet models were then evaluated separately on the testing dataset of each MRI modality for binary image segmentation. The model that achieved the highest F-measure accuracy out of all the four models was selected to detect the ROI in MRI images in the next step. The information for separating different sub-tumor regions (edema, necrosis and enhanced tumor) exists in different MRI modalities. Therefore, three MRI modalities images (FLAIR, T1ce and T2) were combined for segmentation. The ROI images were then generated from the combined MRI modalities based on the obtained ROI mask images, where all the pixels in the combined MRI modalities which correspond to the zero values in the ROI masks are set to zero, while the others are kept unchanged. The generated ROI images were used as the inputs in the next stage of our proposed method to segment sub-tumor structures. See Fig. 3.

**Fig. 4:**
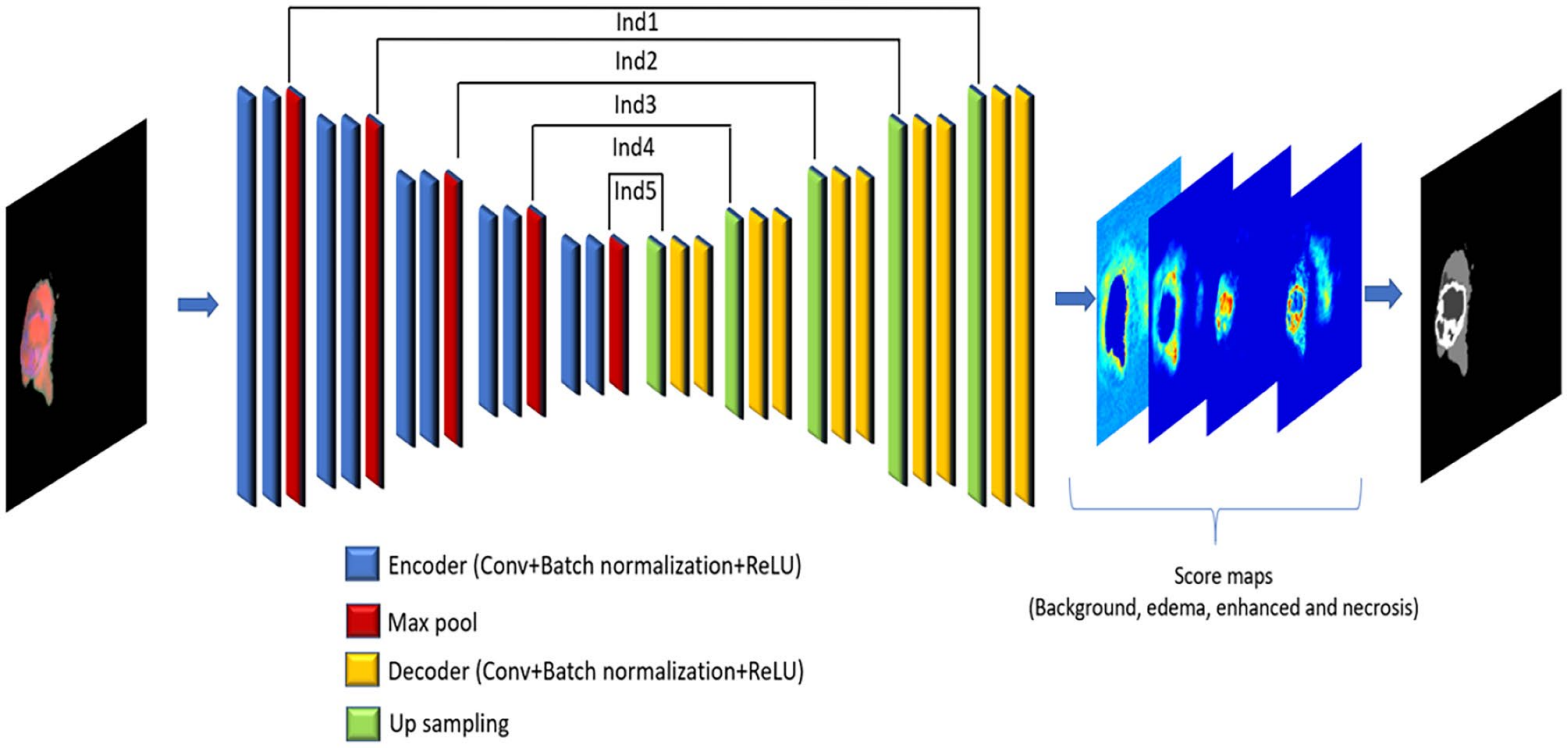
(**a**) Combined MRI modalities, (**b**) ROI masks Images and (**c**) MRI ROI Score maps Features extraction in SegNet network

The semantic segmentation model in Fig. 4 takes full-size images as inputs for feature extraction in an end-to-end way. The pre-trained SegNet is used, and its parameters are tuned using the images with manually annotated tumor regions. In the testing process, the final SegNet model is used to create predicted segmentation masks of tumor regions for unidentified images. The motivation for using SegNet network instead of other deep learning networks is that SegNet has a small number of parameters which does not need high computational resources like DeconvNet [13], and it is easier to train end-to-end. Moreover, in U-Net network [14], the entire feature maps in the encoders are transferred to the corresponding up-sampling decoders and concatenated to the decoder feature maps, which leads to high memory requirement, while in SegNet only pooling indices are reused with less memory.

### Machine Learned Feature Extraction with SegNet

After the ROI MRI images are obtained, we first use them to fine-tune a modified pre-trained SegNet [21] for semantic pixel-wise segmentation of the brain tumor regions (edema, necrosis and enhanced) in the images. In the testing stage, the final segmentation is obtained by max-voting to the final score maps of the SegNet. From experimental comparison of the output results with the ground truth, we found that the SegNet network can successfully segment some parts of different brain tumor regions. However, some of the output segmentation results have label dissimilarity between similar pixels, which leads to decrease of the SegNet performance in brain tumor region segmentation. The reason behind it is that the SegNet is not capable of catching all the changes in the brain tissues that are caused by the tumor. Therefore, we will incorporate information that reflects these tissue changes via using texture features.

The extracted features from the SegNet are the score maps produced from the trained SegNet for each output class. After the last decoder layer in the SegNet, the final predicted segmentation mask was obtained by setting each pixel label as that of the score map with the maximum value among all the final maps. Those score maps contain all the hierarchy’s features that are present in the lower and higher resolutions. It can be seen in Fig. 4 that the number of classification labels is the same as the number of score maps in the case of BRATS 2017 dataset.

A four-dimensional feature vector is created for each pixel in the MRI images. The value of each score map layer is equivalent to the value of each element in the feature vector for the corresponding pixel.

### Hand-Crafted Feature Extraction with GLCM

GLCM-based texture features has the ability to describe different types of regions because different natures of tissues in MR images present different textures. Consequently, the texture descriptors will have enough discrimination power to distinguish among the region types [15]. Fusing GLCM-based texture features with SegNet features can incorporate more powerful feature descriptors into the final segmentation, which can help to overcome the limitation in the SegNet network and improve the performance in brain tumor segmentation.

We extracted GLCM-based texture features and SegNet features from the ROI regions of MR images because we only need to perform segmentation in the ROI regions. In this paper, the GLCMs are constructed in four directions: $\theta = 0^\circ , 45^\circ , 90^\circ$, and $135^\circ$ with pixel distance d=1. GLCM-based texture features are calculated using the built-in function in MATLAB. The GLCM approach can deliver the spatial interrelationships of grey tones which are utilized in optimization of the brain tumor segmentation method.

**Fig. 5:**
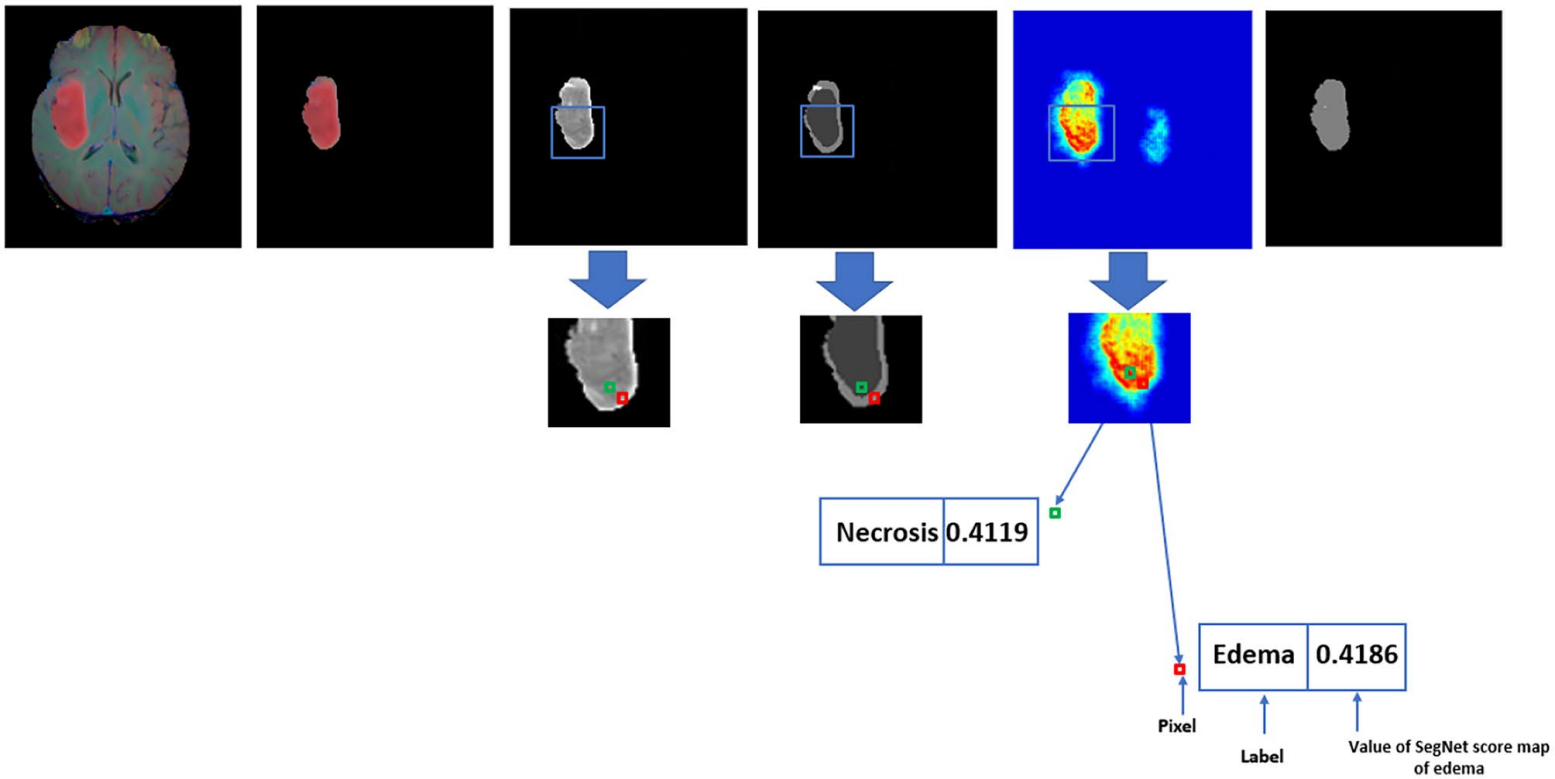
(left to Right): MRI modalities, ROI image, T1ce MRI modality, GT, SegNet score map of edema and predicted mask

### Combined Feature Extraction

**Fig. 6:**
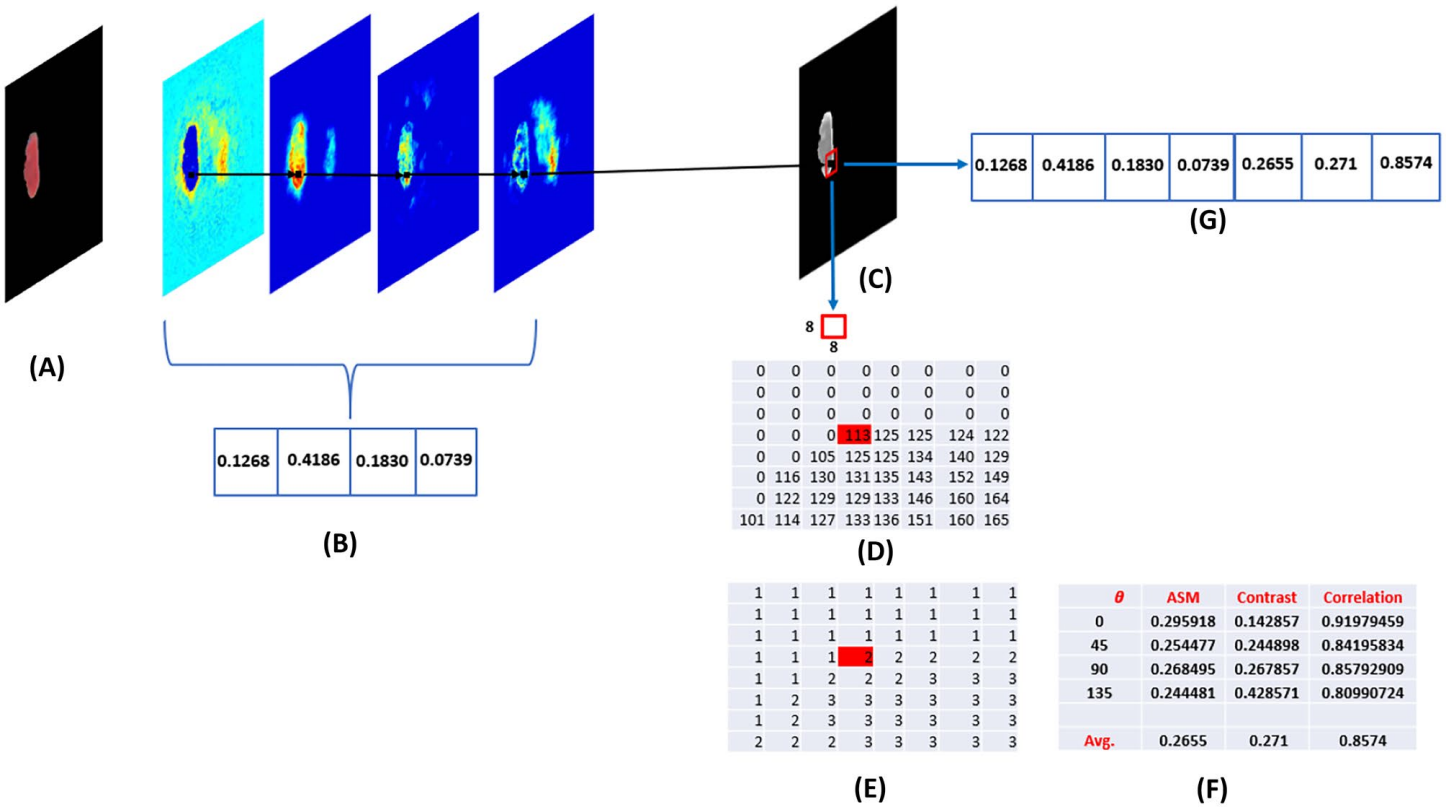
(**A**) ROI image, (**B**) SegNet score maps feature (background, edema, necrosis and enhanced), (**C**) T1ce MRI modality, (**D**) A (${8\times 8}$) T1ce image block of the interest pixel in red color, (**E**) Scaled version of the image block for the same interest pixel, (**F**)Texture features extracted from GLCM spatial dependencies matrices and (**G**) Output feature vector of the corresponding pixel

This section describes spatially combined features which are optimized from SegNet and GLCM-based features. Figure 5 shows an example of patient image, SegNet-based score map of the necrosis class, and the feature representations of two pixels of different classes (edema and necrosis). In the edema score map of the SegNet network, there is no presentation of the obvious separation for necrosis and edema classes. The values for the corresponding pixels are 0.4186 and 0.4119, respectively. These two values are very close. Therefore, the local boundaries of the tumor regions in the edema score map does not have enough detailed presentation. Subsequently, the predicted mask image which is only based on SegNet method does not match the ground truth image. Considering the local neighbourhood dependencies of GLCM texture features, makes the labels, i.e. edema and necrosis more separable. See Fig. 6.

SegNet-based features are selected from each layer of the four score maps for the corresponding pixel. Whereas, GLCM-based texture features are extracted in a fixed-size window of $8\times 8$, centred at that pixel in T1ce images. The T1ce MRI modality was selected because the tumor core has clear boundaries in this modality, which improves the segmentation performance of the tumor core. These texture features are based on the statistics that represents how frequently one grey level will appear with another specific grey level on the image. Three texture features can be extracted from each special dependency matrices of the grey level for distance d=1. The angular second-moment feature (***ASM***) which is a measure of the image homogeneity, the contrast feature which is a measure the amount of local variation in an image and the correlation feature which is a measure of grey-level linear-dependencies [23]. See Fig. 6(F).

The combined feature vector for each pixel consists of seven elements (Four SegNet scores(background, edema, necrosis and enhanced ) and three GLCM features (ASM, contrast and correlations) as shown in Fig. 6(G). It is fed into the DT to classify the pixel.

### DT Parameters and Segmentation

The decision tree (DT) has a flowchart-like tree structure which is used to categorize each pixel into healthy or tumor brain tissues. Each non-leaf node of the tree represents a test on an attribute, and each leaf node represents a class label. A pixel going through the tree will reach a leaf node that represents its class, i.e., healthy or some type of tumor tissue. The procedure is performed based on the feature representation of the pixel. In the training stage, the tree grows into a specified tree depth $D\_tree$. The reason for using DT as classifier in this study is that DT had been proved to have a high performance accuracy in the brain tumor segmentation field [4].

Taking ROI images as input, we only consider each pixel in the tumor target area. A feature vector (i.e. 7 features for each pixel) is extracted and then fed into the DT classifier for training. To select the optimal parameters of the DT classifier, different depths ($D\_tree$) were tested on the BRATS 2017 dataset. The optimal performance accuracy was obtained in fine tree with $D\_tree=100$ which showed better classification accuracy than the medium and coarse tree with depths $D\_tree=20$ and $D\_tree= 4$, respectively.

## Experimental Results

All 285 patient subjects with HGG (210) and LGG (75) in the BRATS 2017 dataset were involved in this study [4, 11]. Basically, 75% of the patients (158 HGG and 57 LGG) were selected to train the deep learning model and 25% (52 HGG and 18 LGG) were assigned as testing set. There are four types of MRI sequences (Flair, T1, T1ce and T2) for each patient. All images have been segmented manually with four rates (4 labels: 0 – background 1 – the necrotic and non-enhanced tumor, 2 – the edema, 4 – the enhanced tumor). The segmentation ground truth for each subject was observed by experienced neuro-radiologists.

The performance accuracy of the proposed model was evaluated on the test set. As a result of practical clinical application, the standard segmentation of the brain tumor structures are grouped into three different tumor regions which are defined by:

- Whole tumor (edema, necrosis and non-enhanced, enhanced tumor).
- Tumor core (necrosis and non-enhanced, enhanced tumor).
- Enhanced tumor.

In each tumor structure, the segmentation results have been evaluated quantitatively using the F-measure.

We conduct experiments to evaluate ROI mask. We compared the performance of implementing each MRI modality to SegNet network for binary segmentation using different models (ROI_FLAIR_SegNet, ROI_T1_SegNet , ROI_T1ce_SegNet and ROI_T2_SegNet). From Table 1, it can been seen that ROI_FLAIR_SegNet model presents a high-performance accuracy result for ROI detection than the other models. The reason for this is that FLAIR modality is considered as a highly effective sequence image which helps to separate the edema region of hyper-intensity from the cerebrospinal fluid (CSF) as the water molecules signal is suppressed in this type of MRI modality [16].

**Table 1:** Results for the binary segmentation of brain tumor on BRATS 2017 dataset

Model	F-measure Complete tumour structure
ROI_Flair_SegNet	**0.86**
ROI_T1_SegNet	**0.74**
ROI_T1ce_SegNet	**0.76**
ROI_T2_SegNet	**0.80**

**Fig. 7:**
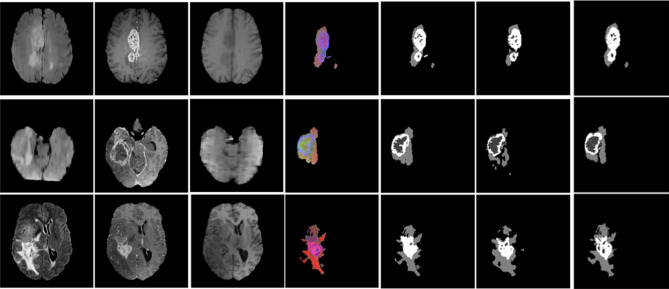
(Left to right) FLAIR, T1ce, T2, ROI images, Ground truth, predicted masks using only SegNet method and predicted masks using SegNet_GLCM_DT method

We conduct another experiment by combining the machine extracted features from the learned SegNet network with the GLCM-based texture features to verify whether the later features help. In Table 2 we can observe that adding the GLCM-based texture features to the model pipeline significantly improves the F-measure performance in the three different brain tumor structures, i.e. whole, core and enhanced tumor. See Fig. 7 for a visual comparison of the segmentation results.

**Table 2:** Comparison of F-measure (mean and standard deviation) for our experiment results separated for whole tumour (WT), tumour core (TC) and enhanced tumour (ET) using BRATS2017 dataset

Methods		F-Measure		
		WT	TC	ET
SegNet	Mean	0.88	0.63	0.6
	Standard deviation	0.03	0.33	0.37
SegNet_GLCM_DT	Mean	0.98	0.75	0.69
	Standard deviation	0.02	0.31	0.35

The reason of better performance from the combined features is that GLCM-based texture features can supply additional textural properties that may be not captured using only SegNet network. Consequently, they can provide local dependencies and neighborhood system of the pixel which is extremely helpful in improving the performance of brain tumor structure segmentation.

Table 3 shows the comparison of our method with some state of the art (SOTA) methods. It can be seen that our method has significantly higher accuracy in whole tumor segmentation than the other SOTA methods. However, this method comes at the expense of having a reduction in core and enhanced tumour segmentation accuracy in comparison to the majority of the other SOTA methods. This is because that the necrosis and enhanced regions have complex structures compared with the edema region, and our method has a relatively low accuracy in detecting necrosis and enhanced tumor. Nevertheless, our method appears to have more accurate results in all the sub-tumour regions than that of [18].

**Table 3:** Performance of our proposed method compared with other methods on BRATS 2017 dataset

Methods	F-measure		
	Whole	Core	Enhanced
Kamnitsas et al. [17]	0.88	0.78	0.72
Wang et al. [19]	0.873	0.783	0.774
Casamitjana et al. [18]	0.86	0.68	0.67
Jiang et al. [27]	0.88	0.83	0.83
Yang et al. [28]	0.90	0.88	0.82
Lin et al. [29]	0.86	0.80	0.77
SegNet_GLCM_DT	**0.98**	0.75	0.69

## Conclusion

This paper proposed a novel method for brain tumor segmentation from MR images. To reduce the computational cost and increase the segmentation accuracy, we proposed to first generate ROI images, which contain only tumor tissues, and then segment the ROI into sub-tumor regions. Considering that the machine-learned features cannot sufficiently represent the tumor tissues in MR images, we combined the machine-learned features generated from a SegNet model with the GLCM-based hand-crafted features, and used a DT to classify each pixel into sub-tumor region based on the combined features. Experimental results showed that FLAIR is the best MRI modality to generate ROI. It was also shown through experiments that the proposed SegNet_GLCM_DT method achieved much better results in whole tumor segmentation compared to some SOTA methods. Specifically, our method achieved F-measure 0.98 in segmenting whole tumor on the BRATS 2017 dataset. Although our method can achieve very high accuracy in segmenting whole tumour (WT), the accuracy of segmenting tumour core (TC) and enhanced tumour (ET) could be further improved. The reason for the relatively low accuracy in TC and ET segmentation is that the necrosis and enhanced tumor regions have more complicated structures in comparison to the edema region, and our technique has sacrificed the accuracy of necrosis and enhanced tumor detection for better whole tumor segmentation. Our future work will investigate more modification methods to improve TC and ET segmentation while keep the best of WT segmentation accuracy.

## Data Availability

BRATS 2017 dataset is used in this work.

## Code Availability

Code is available on request.

## References

[1] Gordillo N, Montseny E, Sobrevilla P (2013). State of the art survey on MRI brain tumour segmentation. Magnetic Resonance Imaging.

[2] Patel M R and Tse V (2004) Diagnosis and staging of brain tumours. In Seminars in roentgenology (Vol. 39, No. 3, p. 347-360). 10.1016/j.ro.2004.05.005 15372749

[3] Bauer S, Wiest R, Nolte LP, Reyes M (2013). A survey of MRI-based medical image analysis for brain tumour studies. Physics in Medicine and Biology.

[4] Menze BH, Jakab A, Bauer S, Kalpathy-Cramer J, Farahani K, Kirby J, Burren Y, Porz N, Slotboom J, Wiest R, Lanczi L (2014). The multimodal brain tumour image segmentation benchmark (BRATS). IEEE Transactions on Medical Imaging.

[5] Pinto A, Pereira S, Correia H, Oliveira J, Rasteiro D M and Silva C A (2015) Brain tumour segmentation based on extremely randomized forest with high-level features. In 2015 37th annual international conference of the IEEE Engineering in Medicine and Biology Society (EMBC) (pp. 3037-3040).IEEE 10.1109/EMBC.2015.7319032 26736932

[6] Pereira S, Pinto A, Alves V, Silva CA (2016). Brain tumour segmentation using convolutional neural networks in MRI images. IEEE Transactions on Medical Imaging.

[7] Havaei M, Davy A, Warde-Farley D, Biard A, Courville A, Bengio Y, Pal C, Jodoin PM, Larochelle H (2017). Brain tumour segmentation with deep neural networks. Medical Image Analysis.

[8] Dong H, Yang G, Liu F, Mo Y and Guo Y, (2017) Automatic brain tumour detection and segmentation using U-Net based fully convolutional networks. In Annual Conference on Medical Image Understanding and Analysis (pp. 506-517). Springer, Cham.

[9] Lai Y, Viswanath S, Baccon J, Ellison D, Judkins A R , and Madabhushi A (2011) A texture-based classifier to discriminate anaplastic from non- anaplastic medulloblastoma. IEEE 37Th Annual Northeast Bioengineering Conference (NEBEC), pages 1-2.

[10] Holli K K, Harrison L, Dastidar P, Wljas M, Liimatainen S, Luukkaala T, hman J, Soimakallio S and Eskola H (2010) Texture analysis of MR images of patients with mild traumatic brain injury. BMC Medical Mmaging, 10(1), p.8. 10.1186/1471-2342-10-8 PMC3161385 20462439

[11] Bakas S, Akbari H, Sotiras A, Bilello M, Rozycki M, Kirby JS, Freymann JB, Farahani K, Davatzikos C (2017). Advancing the cancer genome atlas glioma MRI collectios with expert segmentation labels and radiomic features. Scientific Data.

[12] Niyazi M, Brada M, Chalmers A J, Combs S E, Erridge S C, Fiorentino A, Grosu A L, Lagerwaard F J, Minniti G, Mirimanoff R O and Ricardi U (2016) ESTRO-ACROP guideline target delineation of glioblastomas. Radiotherapy and Oncology, 118(1), pp.35-42. 10.1016/j.radonc.2015.12.003 26777122

[13] Noh H, Hong S and Han B (2015) Learning deconvolution network for semantic segmentation. In Proceedings of The IEEE International Conference on Computer Vision (pp. 1520-1528).

[14] Ronneberger O, Fischer P and Brox T(2015) U-net: Convolutional networks for biomedical image segmentation. In International Conference on Medical Image Computing and Computer-assisted Intervention (pp. 234-241). Springer, Cham.

[15] Bahadure N B, Ray A K and Thethi H P(2017) Image analysis for MRI based brain tumour detection and feature extraction using biologically inspired BWT and SVM. International Journal of Biomedical Imaging, 2017. 10.1155/2017/9749108 PMC5358478 28367213

[16] Liu J, Li M, Wang J, Wu F, Liu T, Pan Y (2014). A survey of MRI-based brain tumour segmentation methods. Tsinghua Science and Technology.

[17] Kamnitsas K, Bai W, Ferrante E, McDonagh S, Sinclair M, Pawlowski N, Rajchl M, Lee M, Kainz B, Rueckert D and Glocker B(2017) Ensembles of multiple models and architectures for robust brain tumour segmentation. In International MICCAI Brainlesion Workshop (pp. 450-462). Springer, Cham.

[18] Casamitjana A, Catnchez I, Combalia M and Vilaplana V (2017) Cascaded V-Net using ROI masks for brain tumour segmentation. In International MICCAI Brainlesion Workshop (pp. 381-391). Springer, Cham.

[19] Wang G, Li W, Ourselin S and Vercauteren T (2017) Automatic brain tumour segmentation using cascaded anisotropic convolutional neural networks. In International MICCAI Brainlesion Workshop (pp. 178-190). Springer, Cham.

[20] Tustison NJ, Avants BB, Cook PA, Zheng Y, Egan A, Yushkevich PA, Gee JC (2010). N4ITK: improved N3 bias correction. IEEE Transactions on Medical Imaging.

[21] Badrinarayanan V, Kendall A, Cipolla R (2017). Segnet: A deep convolutional encoder-decoder architecture for image segmentation. IEEE Transactions on Pattern Analysis and Machine Intelligence.

[22] Alqazzaz S, Sun X, Yang X, Nokes L (2019). Automated brain tumour segmentation on multi-modal MR image using SegNet. Computational Visual Media.

[23] Haralick RM, Shanmugam K, Dinstein IH (1973). Textural features for image classification. IEEE Transactions on systems, man, and cybernetics.

[24] Ghaffari M, Sowmya A and Oliver R (2020) Automated brain tumour segmentation using cascaded 3d densely-connected u-net. In International MICCAI Brainlesion Workshop (pp. 481-491). Springer, Cham.

[25] Ranjbarzadeh R, Kasgari AB, Ghoushchi SJ, Anari S, Naseri M, Bendechache M (2021). Brain tumor segmentation based on deep learning and an attention mechanism using MRI multi-modalities brain images. Scientific Reports.

[26] Daimary D, Bora MB, Amitab K, Kandar D (2020). Brain tumor segmentation from MRI images using hybrid convolutional neural networks. Procedia Computer Science.

[27] Jiang Z, Ding C, Liu M and Tao D (2019). Two-stage cascaded u-net: 1st place solution to brats challenge 2019 segmentation task. In International MICCAI Brainlesion Workshop (pp. 231-241). Springer, Cham.

[28] Yang T, Song J, Li L, Tang Q (2020). Improving brain tumor segmentation on MRI based on the deep U-net and residual units. Journal of X-ray Science and Technology.

[29] Lin M, Momin S, Lei Y, Wang H, Curran W J, Liu T and Yang X (2021). Fully automated segmentation of brain tumor from multiparametric MRI using 3D context deep supervised U-Net. Medical Physics. 10.1002/mp.15032 PMC11752426 34101845

